# Application of hypoxia-mesenchymal stem cells in treatment of anaerobic bacterial wound infection: wound healing and infection recovery

**DOI:** 10.3389/fmicb.2023.1251956

**Published:** 2023-10-05

**Authors:** Elahe Andalib, Mojtaba Kashfi, Golnaz Mahmoudvand, Elaheh Rezaei, Mohamad Mahjoor, Alireza Torki, Hamed Afkhami

**Affiliations:** ^1^Department of Microbiology, School of Basic Sciences, Islamic Azad University Science and Research Branch, Tehran, Iran; ^2^Nervous System Stem Cells Research Center, Semnan University of Medical Sciences, Semnan, Iran; ^3^Cellular and Molecular Research Center, Qom University of Medical Sciences, Qom, Iran; ^4^Department of Medical Microbiology, Faculty of Medicine, Shahid Beheshti University of Medical Sciences, Tehran, Iran; ^5^Student Research Committee, USERN Office, Lorestan University of Medical Sciences, Khorramabad, Iran; ^6^Department of Microbiology, School of Medicine, Ardabil University of Medical Sciences, Ardabil, Iran; ^7^Department of Immunology, Faculty of Medicine, Iran University of Medical Sciences, Tehran, Iran; ^8^Department of Medical Microbiology, School of Medicine, Shahid Sadoughi University of Medical Sciences, Yazd, Iran; ^9^Department of Medical Microbiology, School of Medicine, Iran University of Medical Sciences, Tehran, Iran; ^10^Department of Medical Microbiology, Faculty of Medicine, Shahed University, Tehran, Iran

**Keywords:** mesenchymal stem cell, hypoxia, wound healing, anaerobic bacteria, wound infection

## Abstract

Mesenchymal stromal cells, commonly referred to as MSCs, are a type of multipotent stem cells that are typically extracted from adipose tissue and bone marrow. In the field of tissue engineering and regenerative medicine, MSCs and their exosomes have emerged as revolutionary tools. Researchers are now devoting greater attention to MSCs because of their ability to generate skin cells like fibroblasts and keratinocytes, as well as their distinctive potential to decrease inflammation and emit pro-angiogenic molecules at the site of wounds. More recent investigations revealed that MSCs can exert numerous direct and indirect antimicrobial effects that are immunologically mediated. Collectively, these antimicrobial properties can remove bacterial infections when the MSCs are delivered in a therapeutic setting. Regardless of the positive therapeutic potential of MSCs for a multitude of conditions, transplanted MSC cell retention continues to be a major challenge. Since MSCs are typically administered into naturally hypoxic tissues, understanding the impact of hypoxia on the functioning of MSCs is crucial. Hypoxia has been postulated to be among the factors determining the differentiation of MSCs, resulting in the production of inflammatory cytokines throughout the process of tissue regeneration and wound repair. This has opened new horizons in developing MSC-based systems as a potent therapeutic tool in oxygen-deprived regions, including anaerobic wound infection sites. This review sheds light on the role of hypoxia-MSCs in the treatment of anaerobic bacterial wound infection in terms of both their regenerative and antimicrobial activities.

## Graphical abstract

Brief explanations regarding the effective role of MSCs in wound healing, the resulting infection, and existing therapeutic strategies.

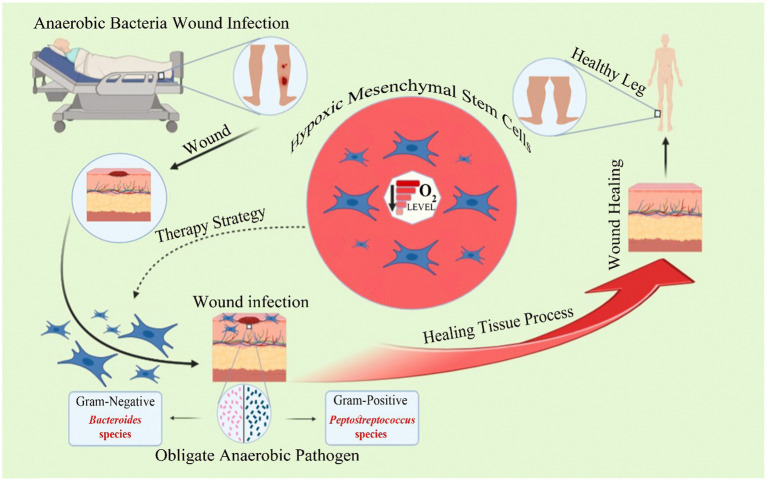

## Introduction

Mesenchymal stem cells (MSCs) are described as stromal cells that possess multipotent, immune-regulatory, and regenerative characteristics. MSCs may originate from bone marrow (BM), adipose tissue, skeletal muscle, dental pulp, and amniotic fluid (Xu et al., 2019; Mahjoor et al., 2021; Nowak-Stępniowska et al., 2022). As a result of the possible therapeutic qualities they offer, MSCs have lately attracted a lot of interest and are increasingly being employed as a therapeutic option for a broad variety of inflammatory immune system disorders (Mahjoor et al., 2023; Wang et al., 2023). In particular, they have emerged as a valuable approach for promoting the healing process of skin wounds (Pulido-Escribano et al., 2023). MSCs have the potential to transform into skin substitutes, thereby acting as a viable alternative to dermal fibroblasts in the process of epidermis generation and skin wound healing. They are capable of differentiating into several cell types in injured areas, including dermal fibroblasts, keratinocytes, and endothelial cells (Hermann et al., 2023; Mahmoudvand et al., 2023; Nilforoushzadeh et al., 2023). Apart from their differential ability, MSCs have additional characteristics, such as being easily harvested and showing minimal immunogenicity. These characteristics, together with their inevitable involvement in the physiology of wound repair, present MSCs as a safe and practical therapeutic method. At the site of injury, MSCs promote the migration of cutaneous cells, angiogenesis, and re-epithelialization, as well as the establishment of granulation tissue, all of which facilitate the process of wound healing (Azari et al., 2022; Bian et al., 2022). The latest evidence confirmed that MSCs can hinder the growth of microorganisms. To provide their profound antibacterial properties, these cells take advantage of both direct and indirect signaling pathways. Directly by constitutively secreted factors and Indirect pathways form by activating the host’s innate immune cells. MSCs can also produce antimicrobial peptides (Leroux et al., 2010). AMPs annihilate bacteria directly by disrupting the integrity of their membrane, or alternatively by inducing the release of proinflammatory cytokines (Leroux et al., 2010; Mahlapuu et al., 2016). A variety of AMPs are released by MSCs, including the cathelicidin peptide LL-37 (Krasnodembskaya et al., 2010), hepcidin (Alcayaga-Miranda et al., 2015), β-defensin 2, and lipocalin 2 (Gupta et al., 2012). These AMPs are regarded as a vital regulator of the capacity of MSCs administered therapeutically to eliminate bacterial infections. MSCs can directly influence the immunological properties of neutrophils and macrophages by secreting PGE_2_ (Vasandan et al., 2016) IL-6, IL-8, and IFN-β, among other factors (Maqbool et al., 2011). Following the exposure to MSC-secreted factors, macrophages acquire an enhanced capacity of phagocytosis, mediated in part by NADPH oxidase activation (Rabani et al., 2018). Neutrophils exposed to MSC conditioned medium are resistant to apoptosis and exhibit a propagated ability of migration (Raffaghello et al., 2008). Studies in animal models of infection have shown that MSCs can increase monocyte recruitment and decrease excessive neutrophil influx as well as neutrophil elastase generation, particularly in mouse models of pulmonary *Pseudomonas aeruginosa* infection and cystic fibrosis (Sutton et al., 2017). Indirect mechanisms involve the recruitment of immune cells and stimulation of macrophages (Chauhan et al., 2023). Macrophages as major parts of the immune system, are implicated in bacterial autophagy and tissue repair (Wang et al., 2023). Under different circumstances, they can develop the anti-inflammatory M2 or the pro-inflammatory M1 phenotypes. Evidence points to the induction of the M2 phenotype by activated allogeneic murine MSCs in infected tissues, while untreated infected areas possess an M1 dominant population. The ability of MSCs to induce the M1 macrophage phenotype is implicated in their anti-bacterial properties. M2 macrophages are assumed to ameliorate the process of wound healing, confirmed by the improved physical and histological appearance of the group that received activated MSCs compared to the other groups (Johnson et al., 2017). MSCs may also propagate alveolar macrophage phagocytosis as shown in a recent *in-vivo* study (Morrison et al., 2017). In a similar way, MSCs promote the recruitment of neutrophils and increase their inflammatory responses in the early stages of bacterial challenge (Brandau et al., 2010). MSCs have emerged as appealing mediators in the treatment of wound infections as a result of these features (Mirshekar et al., 2023).

Notwithstanding the therapeutic advantages of MSCs for wound healing, the cell retention of MSCs after transplantation continues to be exceedingly challenging. MSCs naturally inhabit BM, which has a hypoxic habitat. In addition, the therapeutic delivery of MSCs is often performed in tissues that are hypoxic under normal circumstances. Therefore, several investigations on *in-vitro* cell cultures and subsequent therapeutic applications proposed the cultivation of MSCs under hypoxic conditions (1–10% oxygen; Beegle et al., 2015; Ejtehadifar et al., 2015). Hypoxia is an important factor in the coordination of cell functions, notably controlling the generation of stem cells (Li et al., 2021). Hypoxia-inducing factors (HIFs), which are highly expressed in the presence of diminished oxygen levels, exert different effects on cellular contexts by influencing diverse components of cell biology. Akin to other cell types, activation of HIF-1 elicits a multifaceted response in MSCs within their microenvironment, including alterations in growth, proliferation, differentiation, and gene expression patterns. These effects are mediated by a network of signaling pathways, including Notch and Oct4 (Keith and Simon, 2007; Ejtehadifar et al., 2015).

The normal regenerative process of stem cells is crucial for the replacement of compromised or aging cells with differentiated cells, assisting the proper functioning of various body tissues. Nevertheless, the complete capabilities of these cells have yet to be thoroughly investigated. Several experiments have been conducted to enhance the efficacy of MSCs in promoting therapeutic benefits. Various techniques, including the implementation of hypoxic environments and the isolation of exosomes, were explored. According to a study, MSCs may exhibit resistance to oxygen limitation. Furthermore, hypoxia has the potential to trigger a multitude of stress and survival signaling pathways in MSCs. The research conducted revealed that subjecting MSCs to a hypoxic setting can trigger pathways associated with cellular survival, such as glucose and glutamine metabolism pathways, as well as pathways related to differentiation, growth, and migration (Leroux et al., 2010; Ahmed et al., 2016; Antebi et al., 2018; Lin et al., 2021; Yusoff et al., 2022).

This paper presents a review of the utilization of hypoxia-MSCs (hi-MSCs) for the treatment of anaerobic bacterial wound infections, focusing on both regenerative and antimicrobial mechanisms.

## Anaerobic bacteria

Anaerobic microorganisms are the primary constituents of the indigenous bacterial flora of human mucous membranes and skin and are commonly implicated in endogenous microbial infections (SaiKiran et al., 2022). Anaerobes are challenging to isolate and frequently go unnoticed due to their fastidious nature. Anaerobic microorganisms are frequently detected in mixed infections of both aerobic and anaerobic nature. After the establishment of anaerobic species, there appears to be a phagocytosis obstruction to prevent the decomposition of coexisting microorganisms. Moreover, the transfer of nutrients from one bacterium tends to support the development and spread of another (Negut et al., 2018; Eberly et al., 2022). Infections caused by anaerobic bacteria can arise in sterile areas of the body and pose a significant risk to an individual’s health and well-being (Tjampakasari et al., 2022). Anaerobic infections might appear across many anatomical regions of the body, including but not limited to the central nervous system, oral cavity, chest, abdomen, pelvis, soft tissues, and cutaneous tissue (Brook, 2016).

In regards to wound infections, the existing literature primarily concentrates on facultative or aerobic organisms that are linked to the wound bacterial flora. In contrast, there is limited research that has examined the involvement of anaerobes in chronic wounds (Cheong et al., 2022). The cause of this phenomenon could be attributed to the fact that in the majority of research studies, wounds have been observed over a limited duration, during which anaerobic microorganisms tend to exhibit a slower rate of proliferation compared to their aerobic counterparts (Verbanic et al., 2020). The part played by anaerobic bacteria in wound infection sites is intricate and differs from that of aerobic pathogens. Prolonged surface bacterial colonization may result in venous insufficiency and subsequent colonization of deep tissue layers by anaerobic microorganisms. The probable cause of this condition is the lower oxygen levels in the underlying tissue, which creates a favorable environment for the proliferation of both facultative and obligate anaerobic microorganisms (Finegold, 1993).

The predominant anaerobic bacterial species identified in chronic wounds are *Actinomyces* species, *Bacteroides* species, and *Clostridium* species. In addition, *Peptostreptococcus* species, *Fusobacterium* species, *Finegoldia* species, *Prevotella* species, and *Porphyromonas* species can contribute to wound infection. These organisms usually emerge on the 15th day of the infection process and subsequently exhibit a decline in frequency as time progresses (Nahid et al., 2021). Although particular anaerobic bacteria have been acknowledged for their positive contribution to wound repair, various species have been found to impede the wound-healing process. This is primarily exerted by the production of proteolytic enzymes, stimulation of immune factor release, or suppression of fibroblast and keratinocyte growth (Lindsay et al., 2017; Luqman and Götz, 2021). Table 1 lists anaerobic bacteria that have been found to affect the wound-healing process.

**Table 1:** Anaerobic bacterial pathogens affecting the wound-healing process.

Bacteria species		Mechanism/effector	Effects on wounds	References
*Prevotella*		Possessing a membrane protein that is negatively correlated with cornified envelope (INVO, SPR1A) factors	Disruption of epithelial barrier protein levels	Zevin et al. (2016)
*Bacteroides*	*Ovatus*	Up-regulation of IL-22 expression	Promotion of wound healing	Ihekweazu et al. (2021)
	*Fragilis*	Together with *Escherichia coli*, caused a severe inflammation with massive pus formation in wound	Disrupting the wound healing *via* a synergistic pathway	Kelly (1978)
*Peptostreptococcus*		− ECM degradation *via* the production of proteolytic enzymes including collagenases and aminopeptidases − Inhibition of fibroblast and keratinocyte proliferation, repopulation, and endothelial tubule formation by bacterial supernatant	Prevention of wound healing	Krepel et al. (1992) and Stephens et al. (2003)
*Actinomyces*		Suppression of lymphocyte and fibroblast proliferation through cyclo-oxygenase pathway of arachidonic acid metabolism	Suppression of wound healing	Metzger et al. (1987)
*Clostridium*		Stimulation of keratinocyte cellular responses to injury *via* collagenase	Suppression of wound healing	Riley and Herman (2005)
*Fusobacterium*		Arresting fibroblast growth	Prevention of wound healing	Kapatral et al. (2002)
*Finegoldia*		Resistance to AMPs *via* SufA	Promotion of wound chronicity	Murphy et al. (2014)

IL, interleukin, ECM, extracellular matrix, AMPs, antimicrobial peptides.

## Mesenchymal stem cells

The BM stroma exhibits a structured arrangement of diverse cell types, encompassing stem cells, endothelial cells, adipocytes, fibroblasts, and osteocytes, among others. The two distinct categories of BM stem cells are hematopoietic stem cells and MSCs. Mesenchymal cells can preserve, repair, and restore impaired tissues (Xu et al., 2019). MSCs possess a unique capacity for self-renewing and differentiation. These cells might be extracted from various sources such as the umbilical cord, BM, adipose tissue, endometrial polyps, and menstrual blood (Ding et al., 2011).

MSCs are characterized by the presence of specific markers, including a cluster of differentiation (Vusirikala et al., 2022)_73_, CD_90_, and CD_105_, whereas they lack proteins such as CD_11b_, CD_14_, CD_34_, CD_45_, and CD_79a_ (Andrzejewska et al., 2019). MSCs are capable of generating an abundance of cytokines, including those that facilitate the maintenance of hematopoietic stem cells in their silent phase or promote their self-renewal, such as oncostatin (OSM), stem cell factor (SCF), leukemia inhibitory factor (LIF; Ratcliffe, 2013), stromal cell-derived factor1 (SDF-1), transforming growth factor beta (TGF-β), bone morphogenetic protein4 (BMP-4), and Fms related receptor tyrosine kinase3 (FLT-3). Besides, MSCs release interleukins (IL)-1, 1L-6, IL-7, IL-8, IL-11, IL-12, IL-14, IL-15 (Dazzi et al., 2006).

MSCs generate an extensive list of growth factors, chemokines, and hormones that are involved in various biological processes such as blood vessel formation, immune regulation, and anti-apoptotic functions. MSCs have been observed to augment the process of tissue reconstruction *via* paracrine mechanisms after their transplantation. The protein known as versican plays an integral part in the mechanism of repair. The utilization of MSCs conditioned medium in the cultivation of monocytes can enhance the synthesis of versican protein and may serve as an acceptable substitute for the transplantation of MSCs (Brennan et al., 2020).

## What is a hypoxia condition?

Adenosine 5’triphosphate is synthesized by mammalian cells using the utilization of oxygen and nutrients. The significance of oxygen in numerous biochemical reactions necessitates the preservation the maintenance of oxygen balance. In the presence of hypoxia, cells initiate multiple subsequent cascades, including autophagy, cell stress pathways (e.g., endoplasmic reticulum stress), and energy metabolic pathways [e.g., mTOR complex1 (mTORC1) and HIF-1]. The aforementioned pathways are responsible for the maintenance of homeostasis during periods of hypoxic stress (Ratcliffe, 2013; Nakazawa et al., 2016; Tirpe et al., 2019; Lee et al., 2020). HIFs are a group of heterodimeric factors, comprising HIF-1, HIF-2, and HIF-3 (Tirpe et al., 2019). The mentioned factors consist of a stable β-subunit and an oxygen-sensitive α-subunit. Under normoxic conditions, the separation of the heterodimer results in the hydroxylation of proline residues (proline-402 and proline-564) in the α subunit by prolyl hydroxylases (PHDs; Eales et al., 2016; Dabral et al., 2019), then HIF-1α is ubiquitinated by von-Hippel-Lindau protein (pVHL) enzyme and degraded by the proteasome (Tirpe et al., 2019).

HIFs regulate the transcriptional activation of a multitude of genes that participate in biological processes, including but not confined to cellular growth and proliferation, programmed cell death, cellular metabolism, glycolysis, bacterial infection, and immune system response, as well as tumor formation and spread (Luo et al., 2022).

The normal oxygen concentration in arterial blood is approximately 12%, while its concentration in tissues is 3%. Embryonic stem cells are known to exist under hypoxic conditions, from the point of implantation to fetal growth. In general, during the initial stages of pregnancy, the level of oxygen concentration on the surface of the uterus is in proximity to 2%. Following the development of the placenta, there is a notable elevation in the concentration of oxygen to approximately 8%. Adult stem cells also reside in hypoxic environments in their natural setting. The available evidence indicates that hematopoietic stem cells and BM-derived MSCs (BM-MSCs) coexist within a shared environment (Abdollahi et al., 2011).

Mesenchymal cells can exhibit varying responses to differing concentrations of oxygen, contingent upon their specific microenvironment. For example, the oxygen pressure levels in BM range from 1 to 7%, while in umbilical cord blood and amniotic fluid, they range from 1.5 to 8%. Adipose tissue, on the other hand, exhibits oxygen pressure levels of 10–15%. Under normoxic conditions, it has been predicted that a greater quantity of free radicals is generated, thereby causing interference with the operation of mesenchymal cells. The colony-forming capacity and proliferation rate of MSCs display an augmentation under hypoxic conditions with low oxygen pressure ranging from 1 to 5% (Samal et al., 2021).

## Effects of MSCs on the homeostasis phase of wound healing

Many research projects present evidence that MSCs can boost the coagulation process. It has been revealed that a correlation exists between procoagulant activities and the expression of tissue factors (Mansori et al., 2020), a transmembrane protein that upregulates the production of the thrombin-antithrombin complex (Rangasami et al., 2021). It is noteworthy that extended culture of MSCs results in a boosted expression of TF on the surface of these cells. This may potentially heighten the likelihood of thrombosis (Silachev et al., 2019).

Besides TFs, phosphatidylserine is a pro-coagulation factor that facilitates the synthesis of the thrombin-activating complex by translocating from the inner to the outer layer of the cell membrane. It may be postulated that extracellular vesicles (EVs) obtained from an MSCs-conditioned medium may harbor TF and/or external phosphatidylserine, thereby exerting an influence on blood hemostasis (Silachev et al., 2019). Moreover, the expression of Annexin V on the cellular membrane conduces to the buildup of phosphatidylserine and enhances the process of coagulation (Chance et al., 2019).

### MSCs amend the inflammatory phase

MSCs present at the site of injury determine the immune response of macrophages, neutrophils, and lymphocytes through the secretion of various factors and cytokines. Among these are chemokines (C-C motif) ligand 2 (CCL-2), vascular endothelial growth factor (VEGF), LIF, IL-10, and hepatocyte growth factor (HGF), all of which possess immunosuppressive and regenerative properties. As a result, MSCs enhance the immune system’s defenses against potential infections in wounded tissues (Zhang et al., 2010; Hoang et al., 2020; Silva et al., 2020; Camões et al., 2022).

Besides, MSCs can induce a shift in macrophage polarization from a pro-inflammatory M1 state to an anti-inflammatory M2 state (Ulivi et al., 2014). MSCs have been noted to impede the generation of inflammatory agents from M1 macrophage, including tumor necrosis factor (TNF)-α, while concurrently elevating the levels of TGF-β1 from myofibroblasts (Jiang et al., 2013).

Likewise, MSCs regulate the equilibrium of T helper (Th)1-Th2 cytokines, inducing the synthesis of anti-inflammatory cytokines, among them IL4, while reducing the secretion of the pro-inflammatory interferon-gamma (IFN-γ; Zanone et al., 2010). Finally, MSCs exhibit a suppressive impact on the activity and cytotoxicity of natural killer (NK) cells. The inquiry into the subject reveals that the presence of MSCs results in major alterations in both ligands and receptors that have been proven to facilitate NK cell interactions, along with a reduction in the number of NK cells (Najar et al., 2019).

### Effects of MSCs on the proliferative phase

During the third stage of wound healing, fibroblasts and myofibroblasts play a pivotal role. At this stage, epithelial cells undergo proliferation and restoration, and collagen and other ECM proteins are synthesized. MSCs have been noticed to encourage the growth, migration, and secretion of fibroblasts primarily through the action of platelet-derived growth factor BB (Liu et al., 2022). The utilization of EVs derived from MSCs in a mouse skin burn model resulted in an augmentation of epithelial cell proliferation, thereby promoting wound healing (Zhang et al., 2015; Yates et al., 2017).

MSCs enhance tissue repair by regulating the release of effector T-cell cytokines and switching macrophage polarization to an anti-inflammatory M2 state (Di et al., 2017). *In-vivo*, the expression of CK19, PCNA, and collagen I was all boosted by MSCs. *In-vitro,* exosomes derived from human umbilical cord MSCs triggered the growth of skin cells while protecting them from undergoing apoptosis after heat stress. Exosomes have been confirmed to be enriched in Wnt4, which raises β-catenin nuclear translocation and functioning, thereby promoting skin cell proliferation and migration. This effect could be inhibited by the β-catenin inhibitor ICG001 (Zhang et al., 2015). By manufacturing pro-angiogenic substances, especially VEGFs, epidermal growth factor, C-X-C Motif Chemokine Ligand 12 and HIF-1, MSCs can contribute to the angiogenesis process (Yang et al., 2005; Zhang et al., 2006; Li et al., 2008; Guillamat-Prats, 2021).

### Effects of MSCs on the remodeling phase

The remodeling process is the final stage of wound healing, during which MSCs help coordinate any last-minute changes in the ECM, blood vessels, and resident cells. In particular, MSCs can accelerate wound healing through the secretion of factors related to cell proliferation and differentiation, angiogenic mechanisms, immune suppression, and anti-apoptotic factors (Willer et al., 2022). During this phase, the tissue is subject to regeneration and the collagen fibers undergo organization. MSCs play an active part in the process of matrix remodeling by releasing matrix metalloproteinases (MMPs) to promote the deposition of matrix and secreting tissue inhibitors of metalloproteinases to prevent the deposition of ECM proteins. Evidence confirms that inflammatory cytokines, namely TNF-α, IL-1β, and TGF-β1, propagate MSCs to synthesize MMPs, which in turn triggers the chemotactic migration of MSCs across the extracellular matrix.

It has been found that IL-1β can induce the expression of matrix metalloproteinase-3 (MMP-3) in BM-MSCs. IL-1β can trigger MMP-3 expression *via* ERK1/2, JNK, MAPK p38, and Akt signaling cascades, thereby promoting the migration of MSCs. Stromal cell-derived factor 1 (SDF-1) expressed by MSCs is attributed to the homing ability of MSCs towards the ischemia-induced deteriorated heart muscle tissue. TGF-β1, monocyte chemotactic protein (MCP)-1, TNF-α, and ILs are also believed to boost the migration of MSCs to the injured tissues (Chang et al., 2021).

MSCs are also recognized to emit growth factors and cytokines, including HGF, IL-10, and adrenomedullin, which possess anti-fibrotic traits and aid in the healing of wounds without scarring. HGF and prostaglandin (Arron et al., 2021) E2 generated by MSCs at the site of injury, hinder fibroblast differentiation and help MSCs evade the epithelial-mesenchymal switch. Moreover, classical growth factors and cytokines, such as VEGF, CNTF, GDNF, TGF-β, IL-1β, IL-6, and IL-8, act as paracrine control molecules secreted to extracellular vesicles or exosomes. Recent evidence also insinuates the role of signaling by microRNAs in MSC-derived exosomes (Hofer and Tuan, 2016; Lan et al., 2017).

## MSCs and healing of anaerobic bacterial wound infection in hypoxia condition

Since they were initially identified by Friedenstein et al. (1968) as plastic adherent cells capable of differentiating into different cell lines, MSCs have been broadly investigated for their regenerative characteristics. MSCs exhibit noteworthy regenerative capabilities attributed to their inherent ability to self-renew and differentiate into various tissue types. Recently, there has been a lot of focus on the impact of MSCs on the process of wound repair (Liu et al., 2021). As previously stated, the healing process of skin wounds is intricate and involves various phases, namely homeostasis, inflammation, proliferation, and remodeling. MSCs are known to participate in all phases of the wound-healing process, thereby exerting therapeutic benefits (Mahmoudvand et al., 2023).

On top of that, MSCs have demonstrated robust antimicrobial characteristics *via* both direct and indirect mechanisms (Alcayaga-Miranda et al., 2017).

AMPs are effective in eliminating microbes by disrupting membrane integrity, preventing binding to DNA, and disrupting protein synthesis. AMPs secreted from MSCs can act on fungi, yeasts, and viruses (Mirshekar et al., 2023).

Moreover, MSCs-derived exosomes, which possess antibacterial characteristics, expedite the healing process of diabetic foot ulcers. The exosomes are comprised of biologically active molecules (e.g., nucleic acids, growth factors, and proteins), as well as inactive substances (e.g., antibiotics; Raghav et al., 2021).

Collectively, they alleviate bacterial removal by enhancing the migratory and phagocytic capabilities of neutrophils, which is achieved through the upregulation of IL-6, IL-8, and granulocyte-macrophage colony-stimulating factor levels. These molecules ultimately contribute to the elimination of the infection and promote tissue regeneration, as evidenced in the process of wound healing (Joel et al., 2019). Studies conducted *in-vivo* regarding the antimicrobial properties of MSCs have demonstrated that their transplantation in mice results in the suppression of inflammatory response and the facilitation of bacterial elimination.

The defensin family of AMPs consists of alpha-defensins, β-defensins, and θ-defensins. Defensins are highly implicated in innate and adaptive immunity against microbial and viral pathogens and also contribute to wound healing by upregulating the expression of cytokines and chemokines, producing histamine, and boosting antibody responses. β-Defensins, hBD-1, hBD-2, and hBD-3 are the main functional peptides in humans expressed by many epithelial cells, granulocytes, and MSCs. To date, only one cathelicidin (CAMP) gene has been detected in mice and humans. This gene expresses a protein known as CRAMP, which exhibits a wide spectrum of antimicrobial and anticancer activities as well as chemotactic and antiangiogenic features. It can be detected in several cell types and plays a central part in mucosal defense (Sung et al., 2016).

MSCs also play a crucial role in regulating the immune response and combatting pathogenic microorganisms. This is achieved *via* the production of AMPs that specifically target a range of microorganisms including yeasts, fungi, bacteria, and viruses. MSCs are known to produce several noteworthy AMPs, including LL-37, β-defensin-2, cathelicidin, hepcidin, and lipocalin-2. These peptides are involved in the processes of regeneration, control of proliferation, and migration of MSCs (Gupta et al., 2012; Silva-Carvalho et al., 2022). Similarly, IL-17 and indoleamine-2,3-dioxygenase (Pulido-Escribano et al., 2023) are overexpressed in MSCs. IDO exhibits potent antimicrobial activity against a broad spectrum of bacteria (e.g., *S. aureus*, *S. epidermidis*, Group B *streptococci, and E. faecium*), viral pathogens (*Cytomegalovirus*, *Herpes simplex virus*), and parasitic infections (*Toxoplasma gondii*; Alcayaga-Miranda et al., 2017). The results of an experiment suggested that MSCs can enhance the anti-microbial capacity of equine keratocytes by promoting the expression of AMPs through the secretion of CCL2 (Marx et al., 2021).

The functions of MSCs could potentially be impacted by various environmental factors, including hypoxic conditions. One potential approach to improve the survival of MSCs is to subject them to hypoxic conditions (1–4% oxygen) for a period of 24–48 h before their implantation, which may represent a significant and feasible strategy. MSCs exhibit an up-regulation of HIF-1α in response to pre-exposure to hypoxic conditions (Palomäki et al., 2013). Under the influence of hypoxia, MSCs employ HIF-1α to trigger the AKT signaling cascade, thereby augmenting their growth and survival (Lee et al., 2017). Data from experiments imply that hypoxia preconditioning amplifies the paracrine capacities of MSCs in the context of vascular renewal (Han et al., 2020; Yusoff et al., 2022). The expression of various angiogenic factors, such as VEGF and HGF, is enhanced under hypoxic conditions of 1% oxygen during *in-vitro* culture (Ishiuchi et al., 2020). Hypoxic preconditioning has the potential for amplifying additional traits in MSCs, which may include heightened immunosuppressive properties (Roemeling-van Rhijn et al., 2013), or the synthesis of regenerative growth factors (Chang et al., 2013). Chen et al. (2014) established that under hypoxic conditions, BM-MSCs exhibited elevated expression and secretion levels of basic fibroblast growth factor (bFGF), VEGF-A, IL-6, and IL-8. In addition, the utilization of hypoxic BM-MSCs-derived conditioned medium (hypoCM) in comparison to normoxic BM-MSCs-derived conditioned medium (norCM) resulted in a noteworthy increase in the proliferation of keratinocytes, fibroblasts, and endothelial cells. It also facilitated the migration of keratinocytes, fibroblasts, endothelial cells, and monocytes, along with the production of tubular structures by endothelial cells. The findings of this study indicate that the application of topical hypoCM resulted in a notable acceleration of cutaneous wound contraction in Balb/c nude mice, which is in agreement with the *in-vitro* results. In contrast, the application of norCM or the vesicle control did not produce a similar effect. *In-vivo*, the subjects subjected to hypoCM exhibited a noticeable rise in cell proliferation, neovascularization, and recruitment of inflammatory macrophages. Additionally, a notable decrease in collagen I and collagen III was observed in this group. Similarly, Jun et al. (2014) the study demonstrated that hypoxia had a dual effect on amniotic fluid MSCs (AF-MSCs), as it not only stimulated their proliferation but additionally preserved their inherent features, including surface marker expression and differentiation abilities. It is worth noting that AF-MSCs released a higher number of paracrine factors, specifically VEGF and TGF-β1, into AF-MSCs-hypoCM as compared to AF-MSCs-norCM. The potential of AF-MSCs-hypoCM to improve wound repair has been attributed to its ability to stimulate the production of hypoxia-induced paracrine factors by activating the TGF-β/SMAD2 and PI3K/AKT pathways. The available evidence suggests that hi-MSCs possess augmented capabilities for the process of wound repair (Han et al., 2020).

In a study by Diniz et al. (2016), the utilization of an alginate hydrogel-based delivery system for gingival MSCs exhibited notable antimicrobial efficacy against *Aggregatibacter actinomycetemcomitans*, a type of gram-negative anaerobic bacteria. The antimicrobial activity was dose-dependent, with the highest antimicrobial efficacy being observed at a concentration of 0.50 mg/mL, while concurrently preserving cellular viability. On the contrary, data point to the reciprocal effect of bacterial pathogens on the activities of MSCs. Recent research has shown that the management of the microbiome in wounds can facilitate wound healing (Tang et al., 2023). The analysis of the impact of cell balance between anaerobic bacteria and probiotics on the regenerative properties of MSCs has shown that the simulation of the equilibrium between oral pathogenic bacteria and probiotics using an extract of *Limosilactobacillus reuteri* and *Porphyromonas gingivalis* bacteria can result in bone differentiation and migration of MSCs in controlled laboratory settings. The present study revealed that the combination of *L. reuteri* and *P. gingivalis* has the potential to induce the wound-healing cascade *via* the activation of MSCs (Osakabe et al., 2017; Han et al., 2020). The findings indicate that co-culturing MSCs with anaerobic pathogens in an anaerobic environment provokes the induction of cytokine secretion in the former by the latter, specifically in the anaerobic bacterium *F. nucleatum* (Kriebel et al., 2013). After being co-cultured with *F. nucleatum*, BM-MSCs were found to have the greatest level of IL-8 production, as shown by the results of Biedermann and colleagues. In general, it seems reasonable to depend on hiMSCs in the treatment of anaerobic bacterial wound infection (Han et al., 2020).

## Conclusion

Briefly, MSCs are key cells that mainly originate from BM and adipose tissue. These cells have qualities that are essential for the body, such as the capability of self-regeneration and differentiation. MSCs generate a variety of cytokines, including those that maintain hematopoietic stem cells in the latent phase or drive their self-renewal, such as SCF, OSM, SDF-1, LIF, and BMP-4. TGF-β, which in this manner plays a substantial part in the repair of injuries.

In addition to playing a role in all phases of wound healing, MSCs exhibit strong antimicrobial features. These qualities are manifested in MSCs’ ability to stimulate host innate immune cells and produce AMPs. Because of this, MSCs have shown to be a reliable tool in the fight against bacterial wound infections. MSCs have been proven to be resistant to bacterial infection in anoxic areas because they normally live in a hypoxic microenvironment in the BM. This highlights their potential applicability as an exciting tool in the field of regenerative medicine in areas of the human body that are in contact with anaerobic bacteria. MSCs produced under hypoxia were emphasized in a wide variety of papers for *in-vitro* cell cultures and subsequent therapeutic applications. According to the findings of these investigations, MSCs and anaerobic bacteria have a symbiotic interaction. Delivery systems based on MSCs possess antibacterial characteristics that are effective against anaerobic bacteria. On the other hand, anaerobic bacteria, namely *F. nucleatum*, can cause MSCs to secrete cytokines. When taken together, these results introduce MSCs as a potentially useful tool for wound healing. Nevertheless, despite these advancements, the application of MSCs as a useful instrument in the battle against anaerobic bacterial infections is still in its infancy stage. Subsequent measures needed to be taken to obtain a risk-free MSCs-based strategy for the treatment of wound infections. In this case, verifying the batch release of MSCs-based systems by *in-vitro* assays and assessing their biodistribution and potential adverse effects in pre-clinical studies are of great importance.

## Author contributions

EA and MK: wrote the manuscript. GM, ER, MM, and AT: edited the manuscript and designed figures of the manuscript. HA: design and supervision. All authors contributed to the article and approved the submitted version.
